# Comparison of macrophage responses between high- and low-virulent strain *Edwardsiella tarda* infections in mouse macrophage RAW264.7 cells: interleukin-1β secretion as a key macrophage response to a high-virulent strain *E. tarda*

**DOI:** 10.1128/iai.00663-25

**Published:** 2026-04-21

**Authors:** Xiao-Mi Sun, Mikinori Ueno, Hirofumi Tamaru, Katsuya Hirasaka, Tatsuya Oda, Asami Yoshida, Kiyoshi Osatomi

**Affiliations:** 1 Graduate School of Fisheries and Environmental Sciences, Nagasaki University 639968 , Nagasaki, Japan; 2 Organization for Marine Science and Technology, Nagasaki University 12961, Nagasaki, Japan; 3 Faculty of Fisheries, Nagasaki University 68334, Nagasaki, Japan; University of Pennsylvania Perelman School of Medicine, Philadelphia, Pennsylvania, USA

**Keywords:** *Edwardsiella tarda*, RAW264.7 cells, macrophage infectious responses, interleukin-1β, apoptosis, pyroptosis

## Abstract

*Edwardsiella tarda,* a major facultative intracellular pathogen affecting aquaculture, can cause hemorrhagic septicemia and hemolytic ascites disease in aquaculture fish. It is characterized by the ability to survive and replicate in macrophages. In this study, mouse macrophage cell line RAW264.7 cells were used as a model system to study macrophage responses toward *E. tarda in vitro*. We found that the high-virulent strain NUF251 can survive and multiply in RAW264.7 cells, but the low-virulent strain NUF194 has no such ability. These results were consistent with our previous study conducted in fish macrophages. Both strains induced an increase in intracellular reactive oxygen species and secretion of nitric oxide and tumor necrosis factor-α in RAW264.7 cells, but significantly higher levels of these mediators were induced by NUF194. In addition, DNA fragmentation concomitant with slight activation of caspase-3 was observed in NUF194-infected RAW264.7 cells, suggesting the occurrence of apoptosis, whereas no such clear typical apoptosis symptoms were detected in NUF251-infected RAW264.7 cells. Interestingly, a much higher level of interleukin (IL)-1β was secreted from NUF251-infected RAW264.7 cells as compared to those of NUF194-infected or LPS-treated cells, especially at 3 h post-infection, even though similar levels of IL-1β mRNA were detected in all these cells. Since the secretion of IL-1β is a characteristic feature of pyroptosis, our results suggest that induction of pyroptosis accompanied by IL-1β secretion may be a specific macrophage response linked with the virulence of *E. tarda* infection.

## INTRODUCTION

*Edwardsiella tarda* (*E. tarda*) is a common and serious pathogen with a wide host range, including fish, mammals, reptiles, and humans (1, 2). Edwardsiellosis caused by *E. tarda* has been reported in different fish species, such as Japanese flounder (*Paralichthys olivaceus*), channel catfish (*Ictalurus punctatus*), Japanese eel (*Anguilla japonica*), and yellowtail (*Seriola quinqueradiata*) (3–5). Edwardsiellosis is a generalized septicemia, and signs of the disease may include necrotic abscess, distended abdomen, and swollen anus due to the accumulation of ascitic fluid, and so on (6). This infectious disease causes extensive economic losses in the aquaculture industry worldwide (5). Infection of *E. tarda* as a facultative intracellular pathogen generally activates innate immune host defense mechanisms. In host defense processes, macrophages can produce several mediators, such as reactive oxygen species (ROS), nitric oxide (NO), and proinflammatory cytokines (e.g., TNF-α), which all play vital roles in defense against a broad spectrum of pathogens in fish phagocytes (7–9). However, overproduction of several mediators can often lead to detrimental side effects (such as oxidative stress) and cause damage to the host (10–13).

It has been demonstrated that virulent *E. tarda* can survive and multiply inside fish phagocytes (2, 14), and this vital virulence factor leads to serious infection. Therefore, clarification of the interactions between macrophages and *E. tarda* is crucial to understand the outcome of infection. Fish macrophages are the first choice to study fish host innate immunity against *E. tarda* infection. Unfortunately, there is now no appropriate mature fish macrophage cell line available for *in vitro* studies. The preparation of fish primary cultures from certain tissues is a possible strategy, but terminally differentiated primary macrophages have some drawbacks, such as their scarcity, heterogeneity, and resistance to gene transduction (15, 16). We found that the peritoneal macrophages from Japanese flounder (*Paralichthys olivaceus*) cannot survive more than 24 h and are unable to proliferate. Thus, feasible and practical experiments are limited. A suitable macrophage cell line model system is an effective alternative to solve these problems. RAW264.7, a mouse macrophage cell line, has many advantages, including widespread availability, ease of culture, rapid growth rate, transfection for genetic manipulation, and phenotypic resemblance to primary macrophages (17, 18). Moreover, it has widely been used to study the interactions with bacteria, such as *Pseudomonas aeruginosa* (19), *Aeromonas hydrophila* (20), *Salmonella typhimurium* (21), and *Listeria monocytogenes* (22). Hence, the RAW264.7 cell line might be a good option for studying the interaction between macrophages and *E. tarda*.

Our previous studies have shown that high-virulent (NUF251) and low-virulent (NUF194) strains of *E. tarda* stimulate RAW264.7 cells, via extracellularly secreted factors (ECFs), to induce the production of inflammatory mediators to different extents (12). Further studies have demonstrated that flagellin in ECFs is the main factor responsible for macrophage activation, and the structures of the flagellins are quite different between NUF251 and NUF194 (23, 24). During the studies, we realized that gradual morphological changes in RAW264.7 cells occurred after *E. tarda* infection, suggesting certain pathways of cell damage by programmed cell death (PCD) are progressing in the infected cells. PCD is one of the most intensively studied topics in the field of cell biology. Moreover, increasing evidence shows that PCD is indispensable in many fundamental immunologic processes (25). Three key cell death pathways have been studied: apoptosis, pyroptosis, and necroptosis (26). Apoptosis (27) and pyroptosis (28, 29) play important roles in bacterial infection. For instance, pathogen-infected cells actively kill themselves through PCD before the multiplication of pathogens to prevent the pathogen dissemination (30). Certain PCDs are triggered by Toll-like receptors (TLRs) or Nod-like receptors (NLRs) pathway activation. TLRs are located in the cell surface membrane and endosome and recognize pathogen-associated molecular patterns such as lipopolysaccharide (LPS) and flagellin, via TLR4 and TLR5, respectively (31). NLRs are expressed in the cytosol of immune cells, including macrophages, and recognize intracellular infections (32). For example, NLRC and NLRP play roles in the response to microbial infection and the formation of the inflammasome, respectively. Both receptors recognize pathogens, resulting in the activation of signaling pathways to produce inflammatory cytokines and chemokines to activate the immune system. In addition, during an infection, certain self-reactive immune cells are activated and can negatively impact the host itself. Therefore, some parts of activated immune cells should be eliminated soon after the final stage of infection-related immune responses to protect the host from excessive immune reactions (30). PCD of macrophages is considered to be a strategic response for such self-elimination processes as a host-defense mechanism.

Degradation of nuclear DNA into nucleosomal units is a hallmark of apoptotic cell death. Cleavage of chromosomal DNA, known as apoptotic DNA fragmentation, occurs through a caspase-activation cascade during apoptosis (33). On the other hand, pyroptosis is commonly induced by the gasdermin (GSDM) family of proteins and is accompanied by the release of inflammatory cytokines, such as IL-1β and IL-18 (34). Compared to apoptosis, the extent of DNA damage in pyroptotic cells seems to be relatively low, and it tends to cause a random form of DNA fragmentation (35).

To gain insight into the macrophage responses toward *E. tarda* infection, in this study, we conducted comparative studies between high- and low-virulent *E. tarda*-infected RAW264.7 cell models in terms of production of several mediators and induction of PCD during the infection. The results obtained in this study demonstrated that the cell line RAW264.7 was a useful model, and its behavior was significantly different depending on the virulence of *E. tarda*. Our findings might help understand the role of macrophages in host defense processes during *E. tarda* infection.

## RESULTS

### Intracellular replication of *E. tarda* in RAW264.7 cells

**Fig 1:**
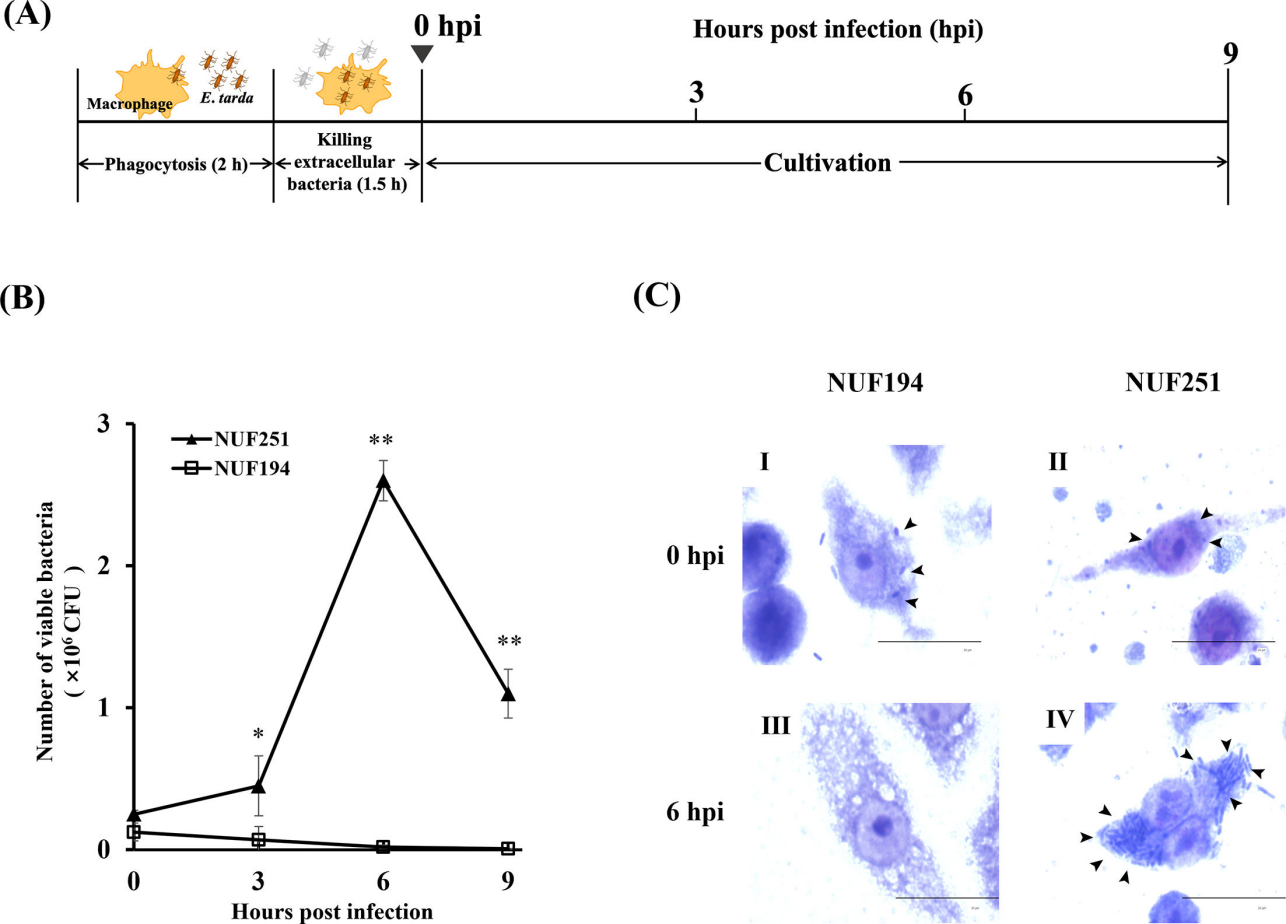
Intracellular replication of *E. tarda* NUF251 and NUF194 in RAW264.7 cells. (**A**) *E. tarda* infection time schedule in RAW264.7 cells. The bacterial cells were added to adherent RAW264.7 cells at a bacteria/macrophage ratio of 10:1 (MOI of 10:1). After 2-h incubation, RAW264.7 cells were treated with gentamicin and washed with PBS to remove the extracellular bacteria, then continued the incubation up to 9 h. (**B**) To determine the number of live bacteria in RAW264.7 cells, the cells were treated with lysis solution containing 1% Triton X-100 at the indicated periods of post-infection times (hpi). The cell lysates were serially diluted with PBS and plated onto nutrient agar and then cultured at 27℃ for 2 days for the formation of bacterial colonies. The results were evaluated by the Student’s *t*-test. Asterisks indicate significant differences between NUF251 and NUF194 (^*^P < 0.05, ^**^P < 0.01). (**C**) *E. tarda*-infected RAW264.7 cells were fixed with 100% methanol and subjected to Giemsa stain, and then observed microscopically. RAW264.7 cells infected with NUF194 (I: 0 hpi, III: 6 hpi) and NUF251 (II: 0 hpi, IV: 6 hpi). Arrows indicate bacterial cells in the cytoplasm of RAW264.7 cells. Each bar = 20 µm. Note that there were many bacterial cells in NUF251-infected RAW264.7 cells (IV), but no bacteria were observed in NUF194-infected cells (III) at 6 hpi.

We compared the ability of intracellular replication between two different virulence strains of *E. tarda* (multiplicity of infection [MOI] of 10:1) in mouse macrophage RAW264.7 cells. First, *E. tarda* was exposed for 2 h and then treated with gentamicin for 1.5 h to kill extracellular bacteria, at which point the 0-h post-infection (hpi) was defined (Fig. 1A). The number of viable cells of NUF251 gradually increased during 9 hpi (Fig. 1B). At 6 hpi, the maximal propagation level was attained and then declined at 9 hpi. On the other hand, the number of viable NUF194 cells detected at 0 hpi gradually decreased along with the incubation time and became almost undetectable at 6 hpi.

The results of Giemsa staining confirmed the intracellular replication of internalized NUF251. At 0 hpi, intracellular bacterial cells of both NUF251 (Fig. 1C II) and NUF194 were observed (Fig. 1C I). Importantly, many NUF251 cells were heavily occupied in the cytoplasm of RAW264.7 cells after 6 hpi (Fig. 1C IV), whereas no NUF194 cells were observed in the RAW264.7 cells (Fig. 1C III). These results indicate that NUF251 can survive and multiply within the RAW264.7 cells, but NUF194 has no such ability.

### Intracellular ROS levels

**Fig 2:**
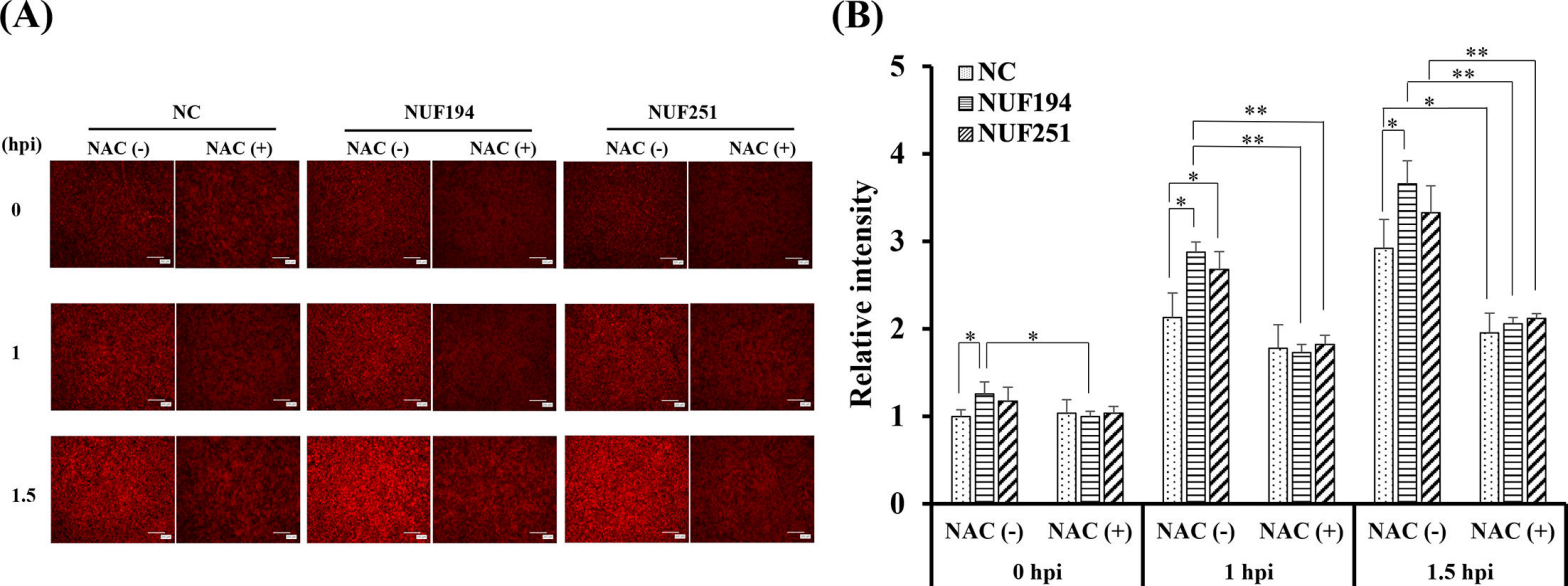
Intracellular ROS levels in RAW264.7 cells infected with *E. tarda* NUF251 and NUF194 in the presence or absence of NAC (final 20 mM). (**A**) After the addition of DHE to the post-infected RAW264.7 cells, fluorescence microscopic observations were carried out at 0, 1, and 1.5 hpi in the presence or absence of NAC. Scale bars indicate 200 μm. (**B**) The fluorescence intensities of the cells were measured with a microplate reader. Each value represents the average of triplicate measurements. Each bar indicates standard deviation. To evaluate significant differences among the test groups, one-way ANOVA was used. Asterisks indicate significant differences between different treatments (^*^P < 0.05, ^**^P < 0.01). NC; normal control cells.

To study intracellular ROS levels in RAW264.7 cells following incubation with each strain of *E. tarda*, fluorescent analysis with a superoxide anion-specific probe (DHE) was conducted. As shown in Fig. 2A, an increase in fluorescence intensity was observed in the cells treated with two strains of *E. tarda* in an incubation time-dependent manner. The relative fluorescence intensities of bacteria-infected cells were statistically higher than the control cells (Fig. 2B). In the presence of an antioxidant, N-acetylcysteine (NAC), a marked decrease in the fluorescence intensities was observed, which confirmed that the fluorescence reflected intracellular ROS levels. ROS levels detected in the normal cells are probably derived from the basal metabolic reactions. Although the differences in the fluorescence intensities between NUF251 and NUF194 at 1 hpi and 1.5 hpi were not statistically significant, NUF194 induced slightly higher ROS production than NUF251 (Fig. 2B).

### NO, iNOS mRNA, and protein levels in RAW264.7 cells infected with *E. tarda*

**Fig 3:**
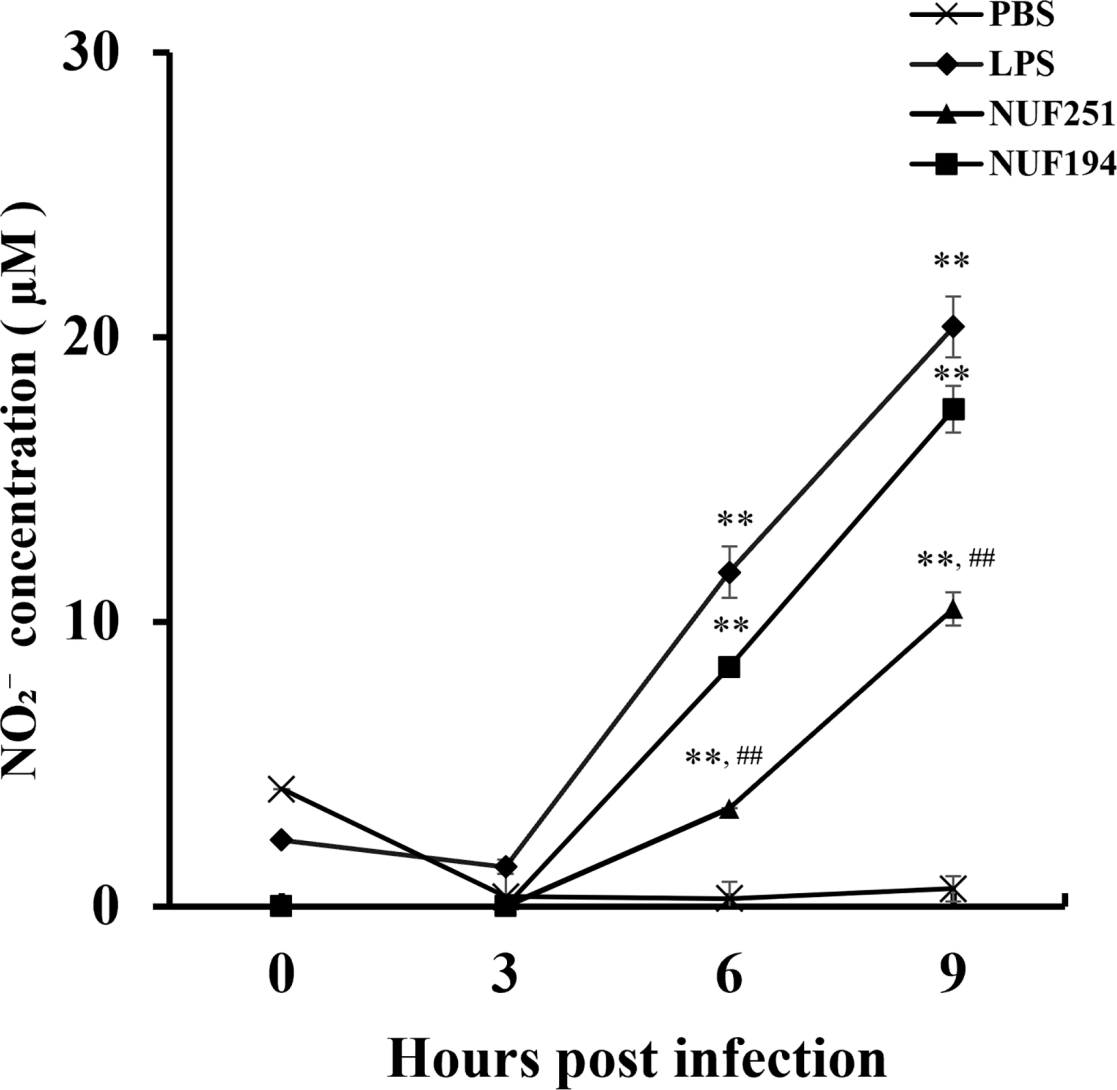
Time-course analyses of NO productions in RAW264.7 cells after *E. tarda* NUF251 or NUF194 infection. The adherent RAW264.7 cells were exposed to each strain of *E. tarda* as the time schedule described in Fig. 1. After the indicated periods of time, the supernatant was withdrawn from each well and subjected to the determination of NO level as described in the text. The cells were also exposed to PBS alone or LPS (final 1 µg/mL) in the same way as bacterial infection. Each value represents the average of triplicate measurements. Each bar represents the standard deviation. The results were evaluated by the Student’s *t*-test. Asterisks indicate a significant difference from the control (**P < 0.01). Hashtags indicate a significant difference between NUF251 and NUF194 (^##^*P* < 0.01).

To examine the responses of RAW264.7 cells against NUF251 and NUF194 infection, the NO levels of the culture supernatants were estimated by the Griess method. As shown in Fig. 3, significant increases in NO production were detected in RAW264.7 cells infected with both bacterial strains, and the values increased along with the incubation times. The NO detected in the *E. tarda*-infected cells was comparable to that observed in LPS-treated cells as the positive control. The NO levels induced by NUF251 were significantly lower than those of NUF194 at 6 and 9 hpi.

**Fig 4:**
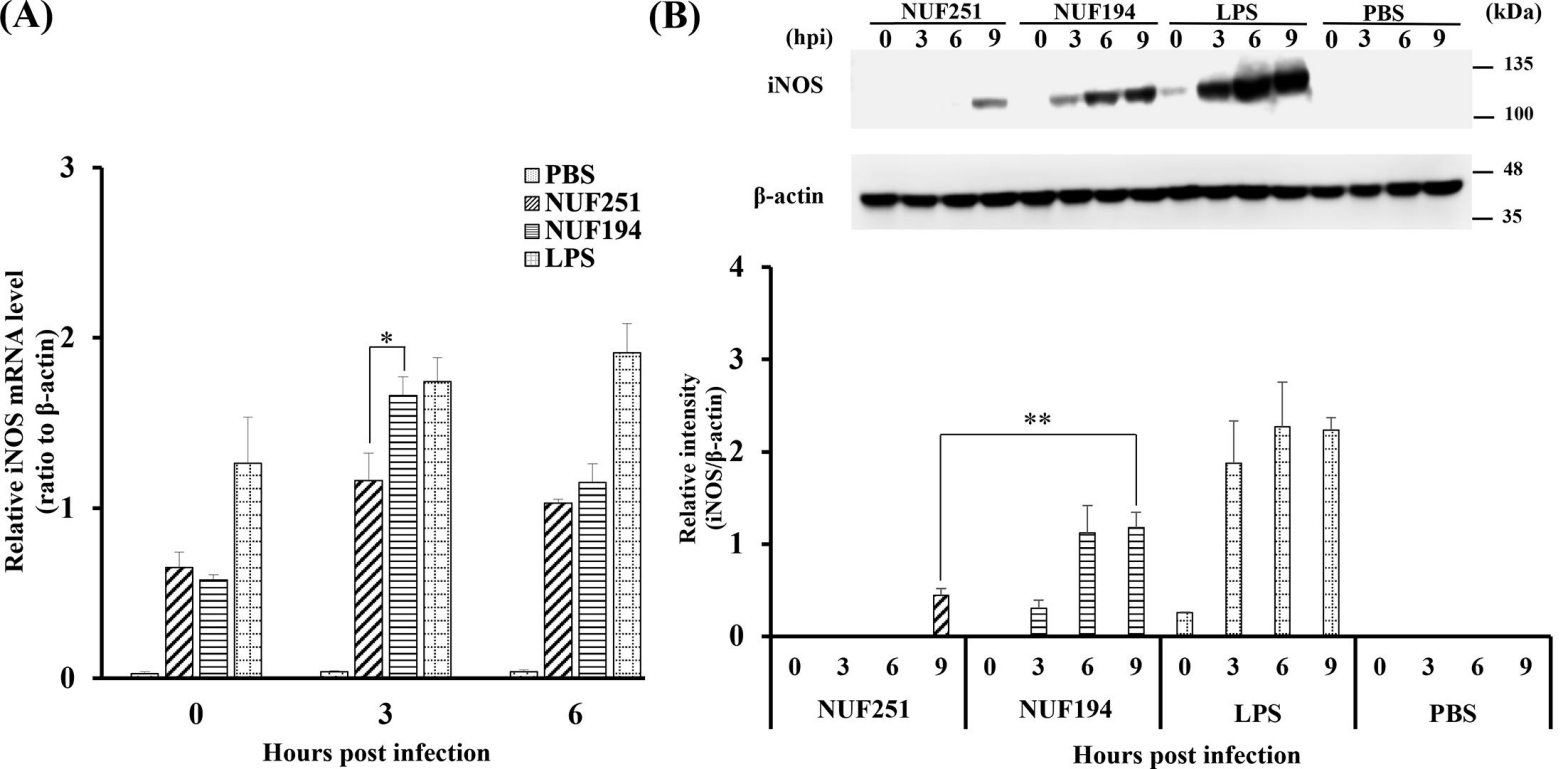
Time-course analyses of mRNA (**A**) and protein (**B**) expression levels of iNOS in RAW264.7 cells after *E. tarda* NUF251 or NUF194 infection. The adherent RAW264.7 cells were exposed to each strain of *E. tarda* as the time schedule described in Fig. 1. (**A**) After the indicated periods of post-infection times (0, 3, and 6 hpi), mRNA levels were determined by qPCR. The values were normalized to β-actin levels as relative mRNA levels. (**B**) After the indicated periods of post-infection times (0, 3, 6, and 9 hpi), iNOS protein expression levels in *E. tarda*-infected RAW264.7 cells were analyzed by western blotting (*n* = 3). The ratio of band intensity was normalized to β-actin levels as relative protein expression levels. The cells were also exposed to PBS alone or LPS (final 1 µg/mL) in the same way as bacterial infection (Fig. 1). Each value represents the average of triplicate measurements. Each bar indicates standard deviation. The results were evaluated by the Student’s *t*-test. Asterisks indicate significant differences between NUF251 and NUF194 (*P < 0.05, **P < 0.01).

To confirm an inducible pathway for NO production from L-arginine catalyzed by inducible NO synthase (iNOS), mRNA and protein levels of iNOS in *E. tarda*-infected RAW264.7 cells were investigated by quantitative real-time PCR (qPCR) (Fig. 4A) and western blot analysis (Fig. 4B), respectively. The results showed that the expression levels of iNOS mRNA and protein increased after infection with the two *E. tarda* strains. Evidently, a higher level of iNOS mRNA was observed in NUF194-infected cells at 3 hpi than that in NUF251, although both were lower than LPS. Regarding the protein levels, the band of iNOS in NUF194-infected cells began to appear at 3 hpi and became thicker thereafter, whereas the band was undetectable until 6 hpi in NUF251-infected cells, and a weak band appeared at 9 hpi (Fig. 4B). These results indicate that both strains of *E. tarda* can induce NO production via iNOS expression with different extents or kinetics, and NUF194 might exert greater macrophage-stimulating activity than NUF251, at least in terms of NO production.

### TNF-α in RAW264.7 cells infected with *E. tarda*

**Fig 5:**
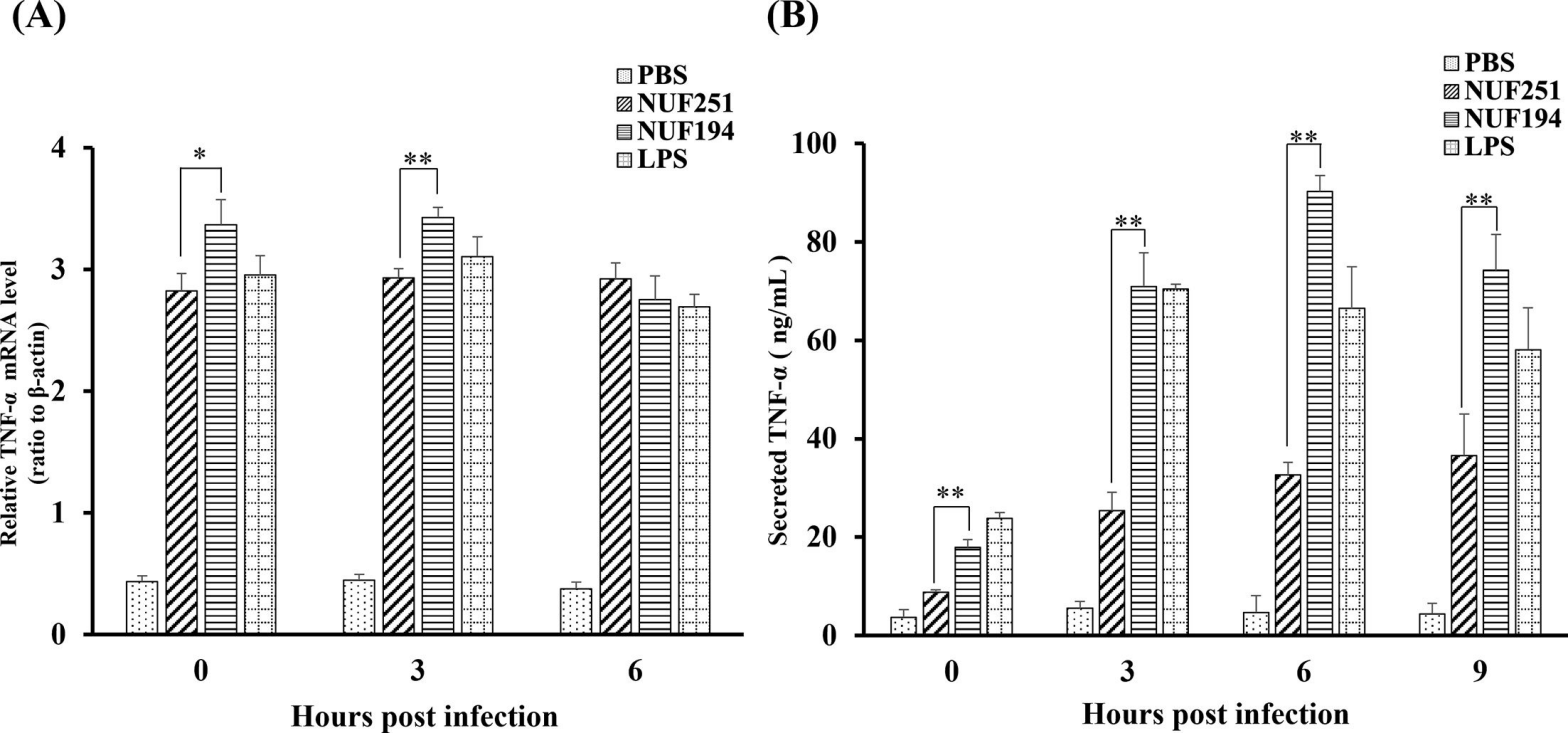
Time-course analyses of intracellular TNF-α mRNA expression (**A**) and extracellular TNF-α secretion (**B**) in RAW264.7 cells after *E. tarda* NUF251 or NUF194 infection. The adherent RAW264.7 cells were exposed to each strain of *E. tarda* as the time schedule described in Fig. 1. (**A**) After the indicated periods of post-infection times (0, 3, and 6 hpi), the TNF-α mRNA levels were determined by qPCR. The values were normalized to β-actin levels as relative mRNA levels. (**B**) The culture supernatants were withdrawn from the wells and subjected to the determination of TNF-α levels by the enzyme-linked immunosorbent assay (ELISA) method as described in the text. The cells were also exposed to PBS alone or LPS (final 1 µg/mL) in the same way as bacterial infection (Fig. 1). Each value represents the average of triplicate measurements. Each bar indicates standard deviation. The results were evaluated by the Student’s *t*-test. Asterisks indicate a significant difference between NUF251 and NUF194 (*P < 0.05, **P < 0.01).

TNF-α mRNA expression in RAW264.7 cells infected with two strains of *E. tarda* was measured by qPCR. As shown in Fig. 5A, the mRNA expression levels of TNF-α in *E. tarda*-infected RAW264.7 cells or LPS-treated cells were remarkably increased as compared to control levels. The values of NUF194 at 0 and 3 hpi were significantly higher than those of NUF251 and became nearly equal at 6 hpi.

The extracellular secretion of TNF-α from RAW264.7 cells after *E. tarda* exposure was determined by the ELISA method (Fig. 5B). Both NUF251 and NUF194 induced TNF-α secretion, but NUF194 induced higher levels of TNF-α with faster kinetics than NUF251. TNF-α secretion of NUF194 was even superior to LPS at 6 and 9 hpi.

### Programmed cell death in *E. tarda*-infected RAW264.7 cells

**Fig 6:**
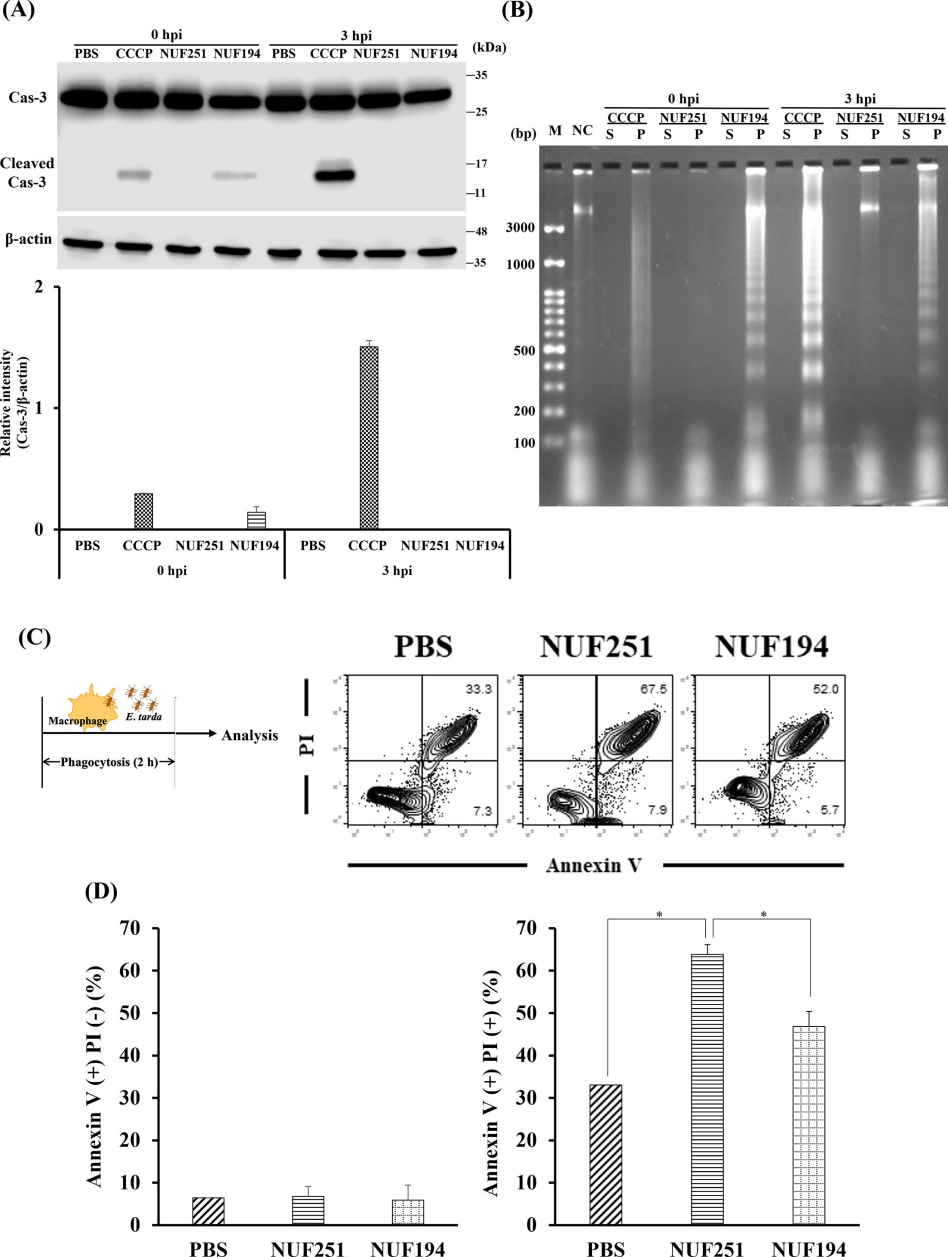
Programmed cell death analysis in RAW264.7 cells after *E. tarda* NUF251 and NUF194 infection (MOI of 50:1). The adherent RAW264.7 cells were exposed to each strain of *E. tarda* as the time schedule described in Fig. 1. (**A**) After the indicated periods of post-infection times (0 and 3 hpi), the cell lysates were prepared and proteins (50 μg/lane) were analyzed for procaspase-3 and cleaved caspase-3 by western blot analysis (*n* = 3) as described in the text. β-actin was used as a loading standard, and the ratio of band intensities of cleaved caspase-3 was normalized to β-actin levels as relative protein expression levels. (**B**) After the indicated periods of post-infection times (0 and 3 hpi), DNA was extracted from the infected cells and analyzed in 2% agarose gel electrophoresis as described in the text. S, supernatant; P, precipitate. M, DNA marker; NC, normal cells as the DNA of untreated RAW264.7 cells. (**C and D**) RAW264.7 cells were exposed to *E. tarda* for 2 h as described in Fig. 1. The cells were detached with a scraper, and then Annexin V-PI staining was performed and analyzed by flow cytometer. In (**D**), each value represents the average of triplicate measurements. Each bar indicates standard deviation. The results were evaluated by the Student’s *t*-test. Asterisks indicate a significant difference between NUF251 and other groups (NUF194 and PBS) (*P < 0.05).

Caspase-3 is a central player in apoptosis progression, and procaspase-3 is converted to an active form by proteolytic cleavage, initiating apoptosis. We analyzed the cleaved caspase-3 in RAW264.7 cells at 0 and 3 h after infection with two strains of *E. tarda* by western blot analysis. In this experiment, CCCP was used as a potent apoptosis inducer as a positive control (Fig. 6A). At 0 hpi, a thin but clear band of cleaved caspase-3 was found in NUF194-infected RAW264.7 cells, as observed in CCCP-treated cells. Although the reason is currently unclear, the band was not detected in NUF194-infected cells at 3 hpi, while the band became thicker in CCCP-treated cells. The band of cleaved caspase-3 was undetectable in NUF251-infected cells at all the incubation times tested, while the presence of procaspase-3 was obvious, as seen in all the treated cells.

To further analyze the programmed cell death in *E. tarda*-infected RAW264.7 cells, a DNA ladder assay was performed. As shown in Fig. 6B, DNA ladder formation was observed in NUF194-infected RAW264.7 cells at 0 and 3 hpi, as seen in CCCP-treated cells, but no clear DNA fragmentation was detected in NUF251-infected cells. These results suggest that NUF194 triggers apoptosis associated with DNA fragmentation and slight caspase-3 activation at an early stage of infection, but NUF251 was incapable of causing such apoptotic symptoms, at least at the time periods tested.

Annexin V-PI staining was conducted to examine whether *E. tarda* induced other apoptotic changes in RAW264.7 cells (Fig. 6C). Generally, adherent cells were detached by trypsin/EDTA treatment, collected, and analyzed using a flow cytometer. However, RAW264.7 cells tightly adhered to the plate of the culture flask are hardly detached by the usual trypsin/EDTA treatment. A previous study reported that detached-RAW264.7 cells prepared using a cell scraper are applicable for flow cytometric analysis with Annexin V assay (36). Therefore, we used a cell scraper to detach *E. tarda*-infected RAW264.7 cells from the plates as mildly as possible to avoid mechanical damage to the cells. There were no significant differences in Annexin V single-positive cell population levels among the tested groups at 2 h of incubation, whereas the Annexin V and PI double-positive populations were 33.0% in the PBS group, 63.8% in the NUF251 group, and 46.8% in the NUF194 group, respectively (Fig. 6D). The value of 33.0% in the PBS group may be due to cell damage caused by the cell scraping process alone. PI can stain the nucleus when the cell membrane permeability is impaired. In apoptotic cells, phosphatidylserine appears on the cell surface as a typical apoptotic response at a relatively early stage. Since Annexin V recognizes phosphatidylserine and binds to it, the Annexin V assay is widely used to detect apoptotic cells. *E. tarda*-infected RAW264.7 cells showed significantly higher Annexin V and PI double-positive cell populations than the PBS group, and the level of the NUF251 group was even higher than that of the NUF194 group (Fig. 6C and D). These results suggest that apoptotic changes are induced in *E. tarda*-infected RAW264.7 cells, and the effect of NUF251 tends to be higher than that of NUF194.

### Secretion of IL-1β from RAW264.7 cells after *E. tarda* infection

**Fig 7:**
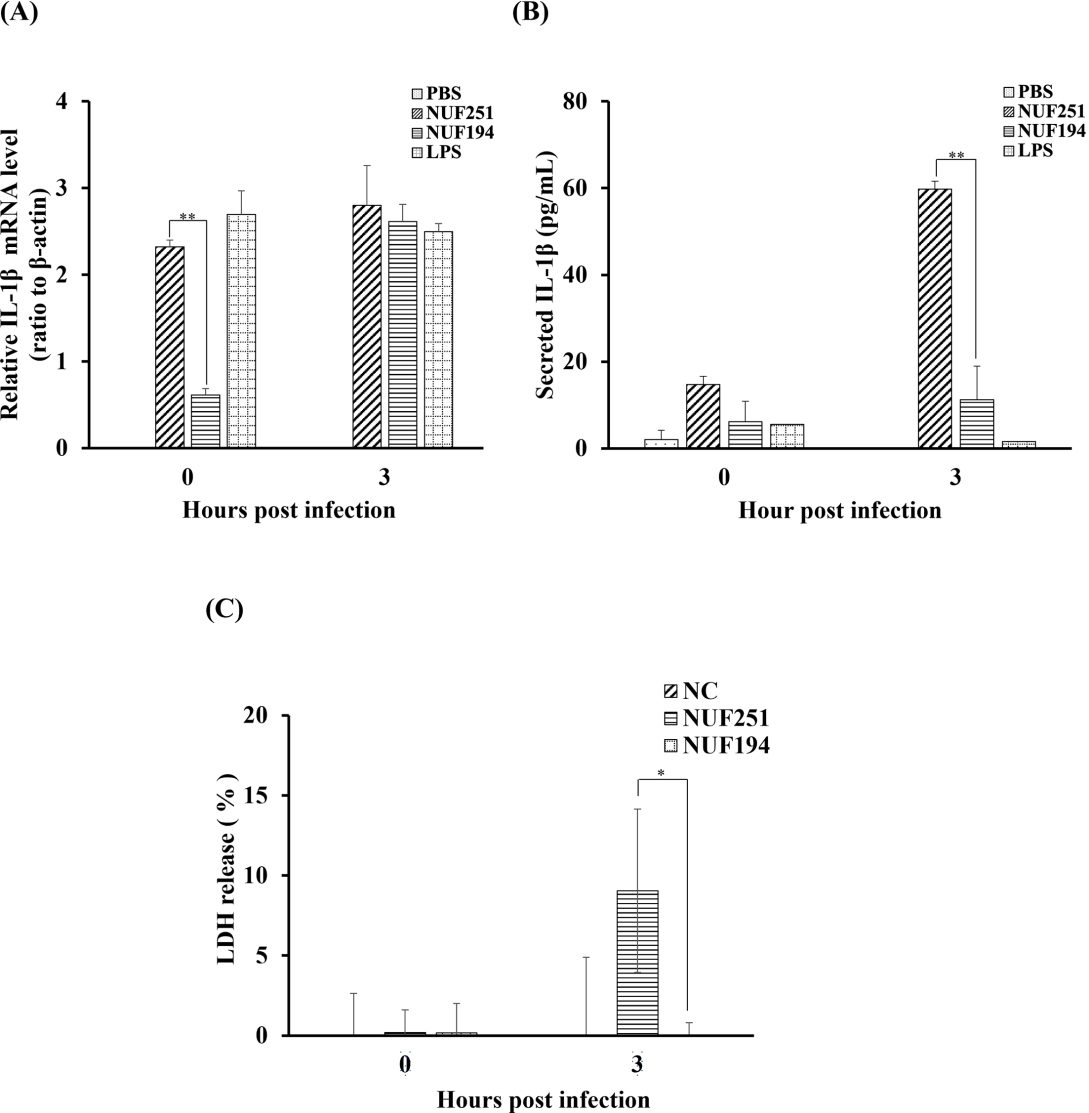
Time-course analyses of IL-1β mRNA expression levels (**A**) and IL-1β secretion (**B**) and lactate dehydrogenase (LDH) release (**C**) in RAW264.7 cells after *E. tarda* NUF251 and NUF194 infection (MOI of 50:1). The adherent RAW264.7 cells were exposed to each strain of *E. tarda* as the time schedule described in Fig. 1. (**A**) After the indicated periods of post-infection times (0 and 3 hpi), the IL-1β mRNA levels were determined by qPCR. The values were normalized to β-actin levels as relative mRNA levels. (**B**) The culture supernatants were withdrawn from the wells and subjected to the determination of the levels of IL-1β by the ELISA method as described in the text. The cells were also exposed to PBS alone or LPS (final 1 µg/mL) in the same way as bacterial infection. Each value represents the average of triplicate measurements. (**C**) An LDH release assay was performed in the culture supernatants of *E. tarda*-infected RAW264.7 cells at the post-infection times (0, 3 hpi). Each value represents the average of triplicate measurements. Each bar indicates standard deviation. The results were evaluated by the Student’s *t*-test. Asterisks indicate a significant difference between NUF251 and NUF194 (*P < 0.05, ^**^P < 0.01).

To analyze other aspects of the cell death mechanism of *E. tarda*-infected RAW264.7 cells, we assessed the expression and secretion of a marker of pyroptosis: IL-1β. IL-1β mRNA in NUF251-infected RAW264.7 cells already reached a quite high level at 0 hpi and then slightly increased at 3 hpi. In the case of NUF194, the IL-1β mRNA level at 0 hpi was lower than that of NUF251 and then increased to a similar level as NUF251 at 3 hpi. In LPS-treated RAW264.7 cells, the expression of IL-1β mRNA was detected at a similar profile with NUF251 (Fig. 7A). The secretion of IL-1β in RAW264.7 cells (after *E. tarda* infection) was measured by the ELISA method. Surprisingly, a much higher level of IL-1β was detected in the supernatant of NUF251-infected RAW264.7 cells, particularly at 3 hpi, which was much higher than even that of LPS-treated cells (Fig. 7B). In NUF194-infected RAW264.7 cells, a much lower level of IL-1β secretion was detected as compared to NUF251, although it was higher than that of LPS-treated cells. The expression and the secretion of IL-1β are probably not directly correlated in *E. tarda-*infected macrophages, and these processes are separately regulated by intracellular signaling mechanisms. These results suggest that the secretion of IL-1β from infected macrophages might be strongly connected with the virulence of *E. tarda*.

Pore formation caused by activated gasdermin D in the cell membrane and subsequent accelerated secretion of intracellular factors through such pores is typically observed in pyroptotic cells. To examine such features of pyroptosis of *E. tarda*-infected RAW264.7 cells, an LDH release assay was performed, in which LDH activities in the cultured supernatants of *E. tarda*-infected RAW264.7 cells were measured. No notable LDH activities were found at 0 hpi in all the groups tested, and only extremely increased LDH activity was detected in the supernatant of NUF251-infected RAW264.7 cells at 3 hpi (Fig. 7C).

## DISCUSSION

In this study, we employed the mouse macrophage cell line RAW264.7 to examine the interaction between *E. tarda* and macrophages in detail. First, we could confirm that both NUF251 and NUF194 bacterial cells could enter RAW264.7 cells. Furthermore, NUF251 cells were able to survive and replicate inside RAW264.7 cells along with the exposure time and reached a maximal propagation level at 6 hpi, while NUF194 had no such ability, and no countable bacterial cells in RAW264.7 cells were detected at 6 hpi (Fig. 1). These results clearly indicate that RAW264.7 cells are suitable for an *in vitro* model system to analyze the interaction between *E. tarda* and macrophages mimicking fish macrophages. Regarding the macrophage cell line model used for *E. tarda* infection, Zhang et al. (37) reported that virulent *E. tarda* cells were incorporated into murine macrophage-like cell line J774A.1, and replicated in the intracellular compartment and reached a peak bacterial cell number after 6-h exposure, similar to our results. Therefore, use of appropriate macrophage cell lines might be the right choice for studying the behavior of *E. tarda* toward macrophages in the short term.

It is widely acknowledged that ROS, NO, and TNF-α are critical mediators produced by macrophages during pathogenic infection as the major macrophage immune responses. For instance, Indian major carp challenged with *E. tarda* exhibited a significantly increased expression of inducible nitric oxide synthase (iNOS) *in vivo* (38). In zebrafish, *E. tarda* infection upregulated the mRNA level of TNF-α (39). Moreover, Ishibe et al. (12) found that *E. tarda* induced NO and TNF-α production from Japanese flounder peritoneal macrophages. Consistent with these findings, both the NUF251 and NUF194 strains were capable of stimulating the RAW264.7 cells to induce NO and TNF-α production (Fig. 3 and 5B) concomitant with the increase in mRNA of iNOS and TNF-α (Fig. 4A and 5A) well beyond control levels. The production levels of NO and TNF-α from RAW264.7 cells exposed to NUF194 were evidently higher than those of NUF251. In addition, fluorescence microscopic observation using ROS-specific probe suggested that NUF194 increased the intracellular ROS level in RAW264.7 cells with slightly greater extent than that of NUF251, which were significantly suppressed with antioxidant NAC (Fig. 2A). Our previous study found that LPS also increased intracellular ROS level in RAW264.7 cells under the similar experimental conditions used in this study (23). These results indicate that two strains of *E. tarda* have a potent ability to stimulate RAW264.7 cells to induce the production of NO, TNF-α, and intracellular ROS. Although the extents of these mediators induced by NUF194 were higher than those of NUF251, it seems unlikely that such differences are sufficient enough to explain the different virulence of the strains. Since LPS generally shows similar stimulating effects on macrophages, the production of these mediators is a common typical response of macrophages often observed during bacterial infections. LPS-like bacterial cell components of *E. tarda* or certain extracellular bacterial products (such as flagellin) might be involved in the macrophage stimulation primarily through the extracellular receptor systems such as Toll-like receptors (23).

Studies investigating host–pathogen interactions have demonstrated that many intracellular bacteria influence the viability of infected cells in different ways. In response to intracellular bacterial pathogens, primarily two types of cell death (such as apoptosis and pyroptosis) may occur (40, 41). Numerous intracellular bacteria elicit apoptosis in macrophages and epithelial cells (27). For instance, *E. tarda* infection induces apoptotic cell death of lymphoid cells in tilapia and causes systemic immunosuppression (42). In contrast, *E. tarda* reportedly replicated in the zebrafish cell line ZF4 and suppressed the apoptosis of *E. tarda*-infected cells (43). Apoptosis of *E. tarda*-infected FG-9307 cells was also prevented, whereas apoptosis of EPC cells was induced by *E. tarda* infection (44). Regarding the mechanisms of suppression of apoptosis by infected bacteria, Okuda et al. (45) showed that *E. tarda* upregulated an anti-apoptosis gene in J774 cells. These findings suggest that *E. tarda* employs different strategies depending on the host cell types through apoptosis induction in macrophages. Although the death of macrophages is a common phenomenon observed during bacterial infection, its biological significance or outcome is complicated and remains controversial. Since the death of macrophages is often associated with the death of infected organisms, it can contribute to the clearance of pathogens and eventual host defense. In contrast, induction of macrophage death can be a pathogen strategy for evading immune cell-mediated host defenses and further dissemination. Under these circumstances, more and more studies are necessary to investigate the interactions between macrophages and bacterial pathogens. To depict the whole picture of macrophage behavior in bacterial infection, even a small piece of information can help.

To gain insights into macrophage responses to bacterial infection, especially focusing on programmed cell death and its biological implications, we investigated the signals reflecting apoptosis or pyroptosis in *E. tarda*-infected RAW264.7 cells. Caspase-3 is a key executioner in many apoptosis inductions. In fact, the band equivalent to the active form of cleaved caspase-3 was detected in RAW264.7 cells infected with NUF194 at 0 hpi (Fig. 6A) as a thinner band than that of CCCP-treated cells, as an apoptosis-positive control. At 3 hpi, the band became undetectable in contrast to the thicker band observed in CCCP-treated cells. In contrast, no clear band of cleaved caspase-3 was detected in NUF251-infected cells at any incubation time. The interpretation of these results still remains unclear, but considering the time-dependent increase in the clear band of cleaved caspase-3 in CCCP-treated cells, it seems obvious that there is no problem with the experimental procedure. These results suggest that programmed cell death pathways are somewhat different between NUF251 and NUF194. In previous studies on apoptosis in bacteria-infected cells, extracellular secretion of caspase-3 has been reported during apoptosis (46). Furthermore, many pathogenic bacteria produce various extracellular factors, some of which inhibit or activate caspase-3 (46). It has been proposed that caspase-3 plays significant roles in bacterial infections other than apoptosis executioner (46). Since *E. tarda* is also known to secrete extracellular factors (23, 24), some of these factors may contribute to *E. tarda*-induced apoptosis in RAW264.7 cells. We have not checked the extracellular secretion of caspase-3 from *E. tarda*-infected RAW264.7 cells in parallel with the effects of extracellular factors of *E. tarda* on apoptosis yet. However, if this is the case, it can lead to missing some part of intracellular caspase-3. Further studies are necessary to clarify these points.

In apoptosis, chromosomal DNA cleavage is induced by DNase activated by caspase, resulting in DNA fragmentation. DNA fragmentation in RAW264.7 cells exposed to NUF194 was detected at 0 and 3 hpi (Fig. 6B) in a similar way to CCCP-treated cells. However, no clear DNA fragmentation was observed in RAW264.7 cells exposed to NUF251 at 0 and 3 hpi. These results suggest that NUF194 might induce caspase-3-dependent apoptosis in RAW264.7 cells during the infection, whereas such NUF251 activity was extremely low or undetectable, at least under the conditions used.

Furthermore, we did an Annexin V-PI staining assay. In Annexin-V-positive cells, phosphatidylserine appears on the cell surface as a characteristic feature of apoptosis, whereas PI-positive cells reflect disorder of membrane integrity with increased membrane permeability. Annexin V and PI double-positive populations in *E. tarda*-infected RAW 264.7 cells were evidently higher than in the PBS group, and the NUF251 group was even higher than that of the NUF194 group (Fig. 6C and D). These results suggest that complex programmed cell death was induced by *E. tarda* infection in RAW264.7 cells, and the effect of NUF251 may be slightly more potent than that of NUF194.

Subsequently, we conducted the experiments focused on IL-1β, which is known to be secreted from macrophages during pyroptosis (47). It was found that NUF251 infection induced the upregulation of IL-1β mRNA in RAW264.7 cells with a similar level of LPS-treated cells (Fig. 7A). The level of IL-1β mRNA induced by NUF194 at 0 hpi was much lower than that induced by NUF251 or LPS and then reached a similar level at 3 hpi (Fig. 7A), suggesting the delayed induction by NUF194. Similar to our results, *E. tarda* infection of macrophages in Japanese flounder also upregulated the IL-1β expression (14). Consistent with our results, LPS stimulation of RAW264.7 cells reportedly resulted in an increase in IL-1β mRNA at 6 h (48). Interestingly, the secretion of IL-1β from RAW264.7 cells infected with NUF251 (especially at 3 hpi) was much higher than others, even though the induced IL-1β mRNA levels in all these cells were almost the same at 3 hpi (Fig. 7B). Since LPS-stimulated RAW264.7 cells reportedly secrete a significant level of IL-1β at 24 h (49, 50), NUF251 can induce the secretion of IL-1β with much faster kinetics than LPS. The exact mechanism of IL-1β secretion from NUF251-infected RAW264.7 cells and the reason for its markedly enhanced secretion remain unclear, but pyroptosis might be relevant as an underlying mechanism. Pyroptosis was originally discovered as caspase-1-dependent programmed cell death of macrophages infected with intracellular pathogens such as *Shigella* and *Salmonella* (40, 41). During pyroptosis, pores are formed in the plasma membranes, and the membrane integrity changes. Subsequently, extracellular release of infected pathogens occurs (40, 41). Enhanced membrane permeability accompanied by pyroptosis might be supported by the fact that significant LDH release was observed in NUF251-infected RAW264.7 cells (Fig. 7C). Furthermore, the decline of the bacterial cell number in the NUF251-infected RAW264.7 cells at 9 hpi (Fig. 1A) may be due to the extracellular escape of infected bacteria by pyroptosis. Before that, IL-1β might be efficiently released from pyroptotic RAW264.7 cells through the pores formed in the plasma membrane. This can explain the reason why IL-1β secretion from LPS-treated cells hardly occurred, probably due to the lack of pyroptosis-inducing ability of extracellular LPS at the early stage. Similar to our study, Zhang et al. (37) detected IL-1β secretion in BMDMs after infection with *E. tarda* EIB202 within 8-h infection, and finally, macrophage pyroptosis is induced in a caspase-1-dependent manner. Thus, secretion of IL-1β is a hallmark of pyroptosis (34). Taken together, these findings, together with our current results, suggest that virulent *E. tarda* might initiate pyroptosis effectively in infected macrophages during intracellular replication, which can contribute to further aggravation of the infectious disease. Regarding this point, Zhang et al. reported that *E. tarda* cells replicated in macrophages escaped to the extracellular space via the induction of macrophage pyroptosis, and the bacterial population released from macrophages acquired enhanced virulence (37). Therefore, induction of pyroptosis of infected macrophages is a bacterial strategy that enables *E. tarda* to enhance the virulence and cause systemic infection by allowing bacteria to evade the host-defense system and enhance the infection.

It has been shown that cytosolic flagellin, recognized by intracellular receptors in *E. tarda*-infected macrophages, stimulates caspase-1 activation, which initiates pyroptosis progression and IL-1β secretion (51). A recent study reported that *E. tarda* infects human macrophages through the type III secretion system and induces IL-1β production and gasdermin D activation (52). Highly virulent bacteria possess type II–IV secretion systems or toxins to enhance their infectious efficiency. Although the exact infection mechanism remains unclear, especially regarding the main virulence factors, a combination of multiple factors may represent a possible mechanism. Our recent study using recombinant flagellins of NUF251 and NUF194 strains revealed that the molecular weights and amino acid sequences of the flagellins of these strains were quite different. In particular, the flagellin of NUF194 has a smaller molecular size, and the equivalent of 57 amino acids was deleted as compared to the flagellin of NUF251 (23). Therefore, we hypothesized that quite different amino acid sequences of flagellin in the two strains reflect NLR responses. Flagellin of NUF251 might elaborate the NLRC4/Pro-caspase-1 complex, which is an inflammasome, resulting in caspase-1 and gasdermin D activation with pore formation in the cell membrane. Thus, one possible speculation is that the difference in the ability between NUF251 and NUF194 to induce IL-1β secretion observed in this study is partly attributable to their different flagellin structures. Further studies focusing on this point might provide insights into the mechanism of IL-1β secretion and pyroptosis induction in *E. tarda*-infected macrophages with regard to its virulence.

Our findings raised further important questions, such as how and why the two strains of *E. tarda* caused different programmed cell death in macrophages, and what is the outcome of macrophage cell death during the entire infection process in terms of the interaction between bacteria and macrophages. These are highly complex issues with multiple perspectives, and we do not currently have enough information to answer these questions. As described above, extracellularly secreted flagellins may play a key role in the induction of intracellular pathways in *E. tarda*-infected macrophages. Further studies focusing on flagellin can provide insights into these issues.

**Fig 8:**
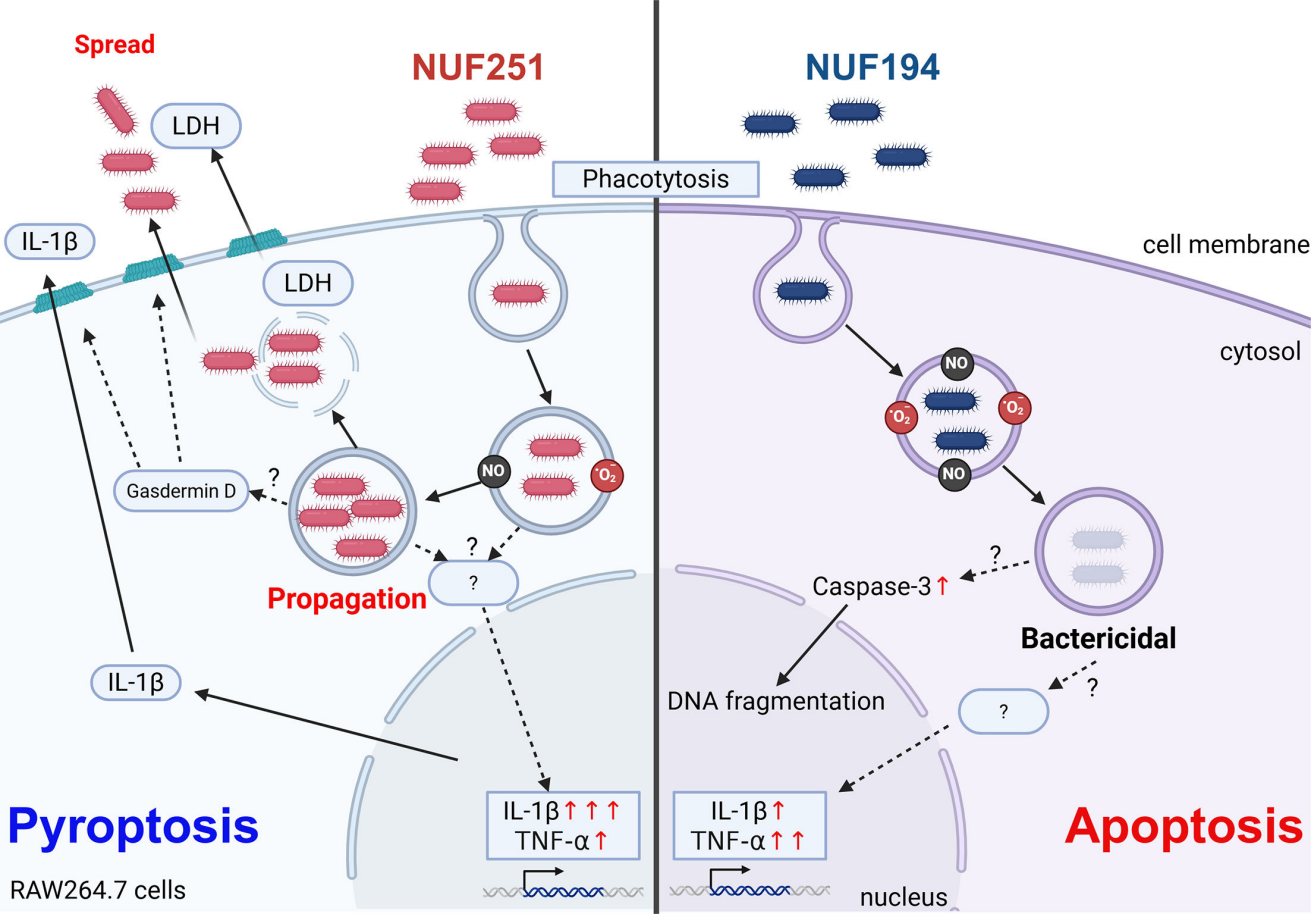
Summary of intracellular signaling pathways induced by the two strains of *E. tarda* infection in RAW264.7 cells. Infection of the two strains of *E. tarda* caused inflammatory responses, such as intracellular ROS production, NO, and TNF-α secretions, to different extents. The activities of NUF194 were slightly higher than those of NUF251. The programmed cell death pathways were also provoked by *E. tarda* as seen in caspase-3 activation, DNA fragmentation, LDH release, and the appearance of Annexin V and PI double-positive cells. These programmed cell death signals differed between the two strains of *E. tarda*. Taking these findings into consideration, potently virulent NUF251 tends to induce pyroptosis, whereas less virulent NUF194 primarily induces typical apoptosis.

In conclusion, we found that RAW264.7 cells closely resembled fish macrophages in their responses to infection by *E. tarda* NUF251 and NUF194 in the short term. Both strains stimulated RAW264.7 cells and induced production of intracellular ROS, NO, and TNF-α, and the activities of NUF194 were greater than those of NUF251. NUF194 triggered caspase-3-dependent apoptosis in infected RAW264.7 cells, while NUF251 showed no such apoptotic symptoms. In contrast, NUF251 infection provoked mainly pyroptosis reflecting an extremely higher level of IL-1β secretion and LDH release from RAW264.7 cells than NUF194 infection (Fig. 7). Our findings obtained in this study were schematically summarized in Fig. 8 to make it easier to understand how the macrophage responses are different between NUF194 and NUF251.

## MATERIALS AND METHODS

### Cells culture and bacterial strains

RAW264.7 cells (mouse macrophage cell line, ATCC number: TIB-71) were purchased from the American Type Culture Collection (Rockville, MD, USA). The cells were cultured in Dulbecco’s Modified Eagle’s Medium (DMEM) (Sigma-Aldrich, St. Louis, MO, USA) supplemented with 10% fetal bovine serum (Gibco, Grand Island, NY, USA), streptomycin (100 μg/mL), and penicillin (100 IU/mL) (Wako, Osaka, Japan) at 37°C in a humidified atmosphere of 5% CO_2_ and 95% air.

In this study, we used two strains of *E. tarda* (strain names and official codes: NUF251 and NUF194) belonging to Nagasaki University, Faculty of Fisheries (NUF), isolated from Japanese flounder intestine in Nagasaki in 1986 and eel pond water in Nagasaki in 1985, respectively. The LD_50_ values of NUF251 and NUF194 to Japanese flounder are estimated to be 4.4 × 10^2^ and >10^9^ CFU/100 g, respectively (53, 54). Based on these findings, we used NUF251 and NUF194 as high- and low-virulent strains, respectively. Two strains were stored at −80°C with 10% glycerol and incubated in a Luria-Bertani (LB) broth medium at 27°C.

### Intracellular multiplication assay and light microscopy

RAW264.7 cells (2.5 × 10^5^ cells/well) were cultured overnight at 37°C. No significant increase in the number of viable cells was observed after overnight culture under the conditions. Each *E. tarda* strain was added to the adherent RAW264.7 cells at an MOI of 10:1, namely at the bacterial and macrophage cells ratio of 10:1, and then incubated for 2 h at 37°C. After gentamicin (final concentration 100 μg/mL) treatment for 1.5 h to kill extracellular bacteria, the cells were washed three times with PBS, and the RAW264.7 cells were then incubated for 0, 3, 6, and 9 h with a low concentration of gentamicin (10 μg/mL) in fresh DMEM. Afterward, 1% Triton X-100 was added to lyse the cells at room temperature for 10 min. The number of viable bacteria in the cell lysates was determined by colony formation assay. Dilution series of the lysates were plated onto LB agar plates, and the number of colonies formed was counted after 2 days of culture at 27°C.

For the light microscopic observation, adherent RAW264.7 cells were infected with *E. tarda* at an MOI of 50:1 and treated with the same procedure as described above. After the indicated periods of time, the cells on the chamber slide were fixed with 100% methanol and stained with Giemsa staining solution (Wako), and then the cells were viewed on a fluorescence microscope (BZ-X810, Keyence, Osaka, Japan).

### Measurement of intracellular O_2_^−^ level

RAW264.7 cells (1 × 10^5^ cells/well) were incubated overnight at 37°C. The adherent cells were treated with *E. tarda* (MOI of 10:1) as described above in the infection protocol section, and then the *E. tarda*-infected cells were incubated with 5 μmol/L dihydroethidium (DHE) at 37°C for 0, 1, and 1.5 hpi. At the same time, *N-*acetylcysteine (NAC), an antioxidant, was added to confirm the ROS-mediated reaction of DHE. The fluorescence intensities of the treated cells on the plates were measured at excitation wavelengths of 518 nm and emission wavelengths of 606 nm with a microplate reader (BioTek Cytation 3, Winooski, VT, USA) or observed under a fluorescence microscope (BZ-X810, Keyence, Osaka, Japan).

### Nitrite assay for estimation of NO

Adherent RAW264.7 cells were infected with *E. tarda* (MOI of 10:1), as described above, and then the cells (1 × 10^5^ cells/well) were incubated with a low concentration of gentamicin for 0, 3, 6, and 9 h at 37°C. After the incubation, the concentration of NO in the culture medium was determined by the Griess method. Briefly, a NO standard curve was prepared for serial twofold dilution of sodium nitrite, and the supernatants of the samples were transferred to a new 96-well plate. Then, the Griess reagent was added to the wells and incubated for 20 min under shaking at room temperature. The absorbance of each well was measured at 550 nm using a microplate reader (BioTek Cytation 3). Lipopolysaccharide (*Escherichia coli* 026:B6; LPS) purchased from Sigma-Aldrich (MO, USA) was used as the comparable control at 1 µg/mL.

### ELISA for TNF-α and IL-1β

TNF-α levels in the samples were measured by the sandwich ELISA method as previously described (24). In this assay, anti-mouse TNF-α monoclonal antibody (Invitrogen, MM350C), F96 Maxisorp Nunc-Immuno plate (Thermo, Roskilde, Denmark), and anti-mouse TNF-α polyclonal antibody (Invitrogen, P350) were used.

The IL-1β levels in the culture supernatants of *E. tarda*-infected RAW264.7 cells at an MOI of 50:1 were measured by the method described previously using the ELISA Assay Kit (R&D Systems, Inc [Minneapolis, MN, USA]) following the manufacturer’s instructions (49). The effect of LPS (final 1 µg/mL) was examined at the same time as a comparable control.

### Total RNA isolation, cDNA synthesis, and qPCR

Total RNA was isolated from the RAW264.7 cells treated as described above using ISOGEN II (Nippongene, Tokyo, Japan) following the manufacturer’s instructions. To synthesize cDNA, total RNA (500 ng) was subjected to reverse transcription with oligo-dT primer CDS-BR (5′-GGC CAC GCG TCG ACT AGT AC(T)_16_-3′), random primer (5′-NNN NNN NNN-3′), and M-MLV reverse transcriptase (Promega, Madison, WI, USA). Primers for mouse iNOS (5′-TCATGACATCGACCAGAAGC-3′ and 5′-TTCGGACATCAAAGGTCTCAC-3′), mouse TNF-α (5′-ACTGAACTTCGGGGTGATCG-3′ and 5′-GGCTACAGGCTTGTCACTCG-3′), mouse IL-1β (5′-CTCATCTGGGATCCTCTCCA-3′ and 5′-GGGTCCGTCAACTTCAAAG-A-3′), and β-actin (5′-TACCACAGGCATTGTGATGG-3′ and 5′-ACGCTCGGTCAGGATCTTC-3′) were used. The qPCRs were performed using a Power SYBR Green PCR Master Mix and a 7300 RT-PCR system (Applied Biosystems, Foster, CA, USA) in accordance with the manufacturer’s instructions. Thermocycling was performed as follows: 10 min at 95℃, 15 s at 95℃, and 1 min at 60℃ for 40 cycles. All data were normalized with β-actin as an internal reference. The effect of LPS (final 1 µg/mL) was examined at the same time as a comparable control.

### Immunoblotting

The detection of iNOS proteins in *E. tarda*-infected RAW264.7 cells was conducted with immunoblotting as previously described (55). In brief, the adherent cells (2 × 10^6^ cells/well) treated with *E. tarda* (MOI of 10:1) were washed three times with ice-cold PBS and homogenized in a 50 mM Tris-HCl, 150 mM NaCl, 5 mM EDTA, 1% Triton X-100, 100 μM MG-132, and proteinase inhibitor cocktail (Nacalai Tesque, Kyoto, Japan) by sonication. The protein concentrations in the cell homogenates were quantified by Pierce BCA assay (Pierce, Rockford, IL, USA). The protein samples were mixed with equal volumes of 2× SDS sample buffer (4% SDS, 100 mM Tris-HCl, pH 6.8, 20% glycerol), and incubated at 95°C for 5 min, then subjected to SDS-PAGE with 20 μg/lane. The proteins were transferred to a polyvinylidene difluoride (PVDF) membrane. The membranes were blocked with 5% skim milk in PBS-0.1% Tween 20 (PBST). Subsequently, the proteins were probed with primary antibodies according to the manufacturer’s instructions. Anti-β-actin (Abcam, ab8227, Cambridge, UK) and anti-iNOS/NOS II (Sigma, 06-573, MO, USA) were used at 1:2,000, respectively. Anti-rabbit IgG, HRP-conjugated antibody as the secondary antibody was used at 1:5,000 (Cell Signaling Technology, #7074, Danvers, MA, USA). The protein bands on membranes were detected by C-DiGit Blot Scanner (LI-COR Biotechnology, Nebraska, USA) using ImmunoStar Zeta Western Blotting Detection Reagents (Fujifilm Wako Pure Chemical Corporation, Osaka, Japan). The intensities of protein bands on the membrane were estimated by Image J software (National Institutes of Health, Bethesda, MD, USA). The effect of LPS (final 1 µg/mL) was examined at the same time as a comparable control. For the detection of caspase-3, RAW264.7 cells were infected with *E. tarda* at an MOI of 50:1, and cell homogenates were mixed with equal volumes of 4× SDS sample buffer (8% SDS, 250 mM Tris-HCl, 40% glycerol, 8% beta-mercaptoethanol, and 0.02% bromophenol blue).

After a 5-min incubation at 95°C, the samples were subjected to SDS-PAGE with 50 μg/lane. The proteins were transferred to a PVDF membrane. Then, the membrane was blocked with 5% skim milk in TBS-0.1% Tween 20 (TBST). Anti-β-actin (Gene Tex, AC-15, Irvine, CA, USA) and anti-caspase-3 antibodies (Cell Signaling Technology, #9662, Danvers, MA, USA) were used at 1:1,000, respectively. Donkey anti-rabbit IgG at 1:5,000 (GE, Healthcare, Little Chalfont, UK) and sheep anti-mouse IgG at 1:5,000 (GE, Healthcare, Little Chalfont, UK) were used as secondary antibodies, respectively. The detection and quantification of protein bands on the membranes were carried out as described above.

### DNA ladder assay

Infections of RAW264.7 cells with *E. tarda* were performed as described above at an MOI of 50:1. Carbonyl cyanide m-chlorophenylhydrazone (CCCP) was used as a positive apoptosis inducer. The treated cells were washed with PBS and lysed with lysis buffer (10 mM Tris-HCl, 10 mM EDTA, 0.5% TritonX-100, pH 7.4) at 4°C for 10 min. Next, the insoluble fractions were pelleted by centrifugation (KUBOTA 3740, model of rotor: AF-2724A) at 20,630 × *g* for 5 min at 4°C, and the supernatants were collected. The supernatants and precipitated fractions were treated with 250 μg/mL proteinase K (Kanto Chemical, Tokyo, Japan) for 2 h at 55°C to remove the proteins. After the treatment with 100 μg/mL RNase A, DNA fractions were precipitated with 5 M NaCl (20 μL) and isopropanol (120 μL) at −20°C overnight. The DNA precipitates were harvested by centrifugation at 20,630 × *g* for 15 min at 4°C. After removing the supernatant, DNA was dissolved in nuclease-free water and subjected to 2% agarose gel electrophoresis.

### Annexin V-PI staining assay

Adherent RAW264.7 cells in 35 mm diameter dishes (2 × 10^6^ cells/well) were exposed to *E. tarda* for 2 h at 37°C as described above in the infection protocol section. After incubation, the cells were washed with PBS. To prepare cell suspensions, the cells were detached with a Falcon cell scraper. The cell suspensions were incubated with FITC-Annexin V (BioLegend, 690905, San Diego, CA) and propidium iodide (PI) for 15 min at room temperature in the dark. The stained cells were analyzed using On-chip Sort (On-chip Biotechnologies Co., Ltd., Tokyo, Japan). The data were analyzed using FlowJo software version 10.8.1 (Tree Star, Carlos, CA).

### LDH release assay

Infections of RAW264.7 cells with *E. tarda* were performed as described above at an MOI of 50:1. After the indicated periods of time, LDH activities in the cultured supernatants of *E. tarda*-infected RAW264.7 cells were measured by Cytotoxicity LDH Assay Kit-WST (Dojindo Laboratories Co., Ltd., Kumamoto, Japan) following the manufacturer’s protocol.

### Statistics

Statistical analysis was conducted by the Student’s *t*-test or Tukey’s multiple comparison post hoc analysis following one-way ANOVA. The criteria of significance were set at ^*^P < 0.05 or ^**^P < 0.01. All values obtained were expressed as mean ± SD.
